# Voluntary physical activity in early life attenuates markers of fatty liver disease in adult male rats fed a high-fat diet

**DOI:** 10.1017/S0007114522002562

**Published:** 2023-05-28

**Authors:** Farqad Abdulqader, Lennex Yu, Mark H. Vickers, Elwyn C. Firth, Sue R. McGlashan

**Affiliations:** 1 Department of Anatomy and Medical Imaging, University of Auckland, Auckland, New Zealand; 2 Te Ira Kāwai – The Auckland Regional Biobank, University of Auckland, Auckland, New Zealand; 3 The Liggins Institute, University of Auckland, Auckland, New Zealand

**Keywords:** Paediatric fatty liver disease, Non-alcoholic fatty liver disease, Exercise, High-fat diet, Fatty liver, Hepatic steatosis

## Abstract

Paediatric fatty liver disease (FLD) can develop into steatohepatitis, cirrhosis and hepatocellular carcinoma in adulthood. We assessed if early life physical exercise reduced the effects of high-fat (HF) diet-induced steatosis. Male HF-fed rats with access to a running wheel from weaning until day (D)60 (early exercise) or from D67 to D120 (late exercise) were compared with control HF- or chow-fed groups with no wheel. At D63 and D120, liver histopathology (Kleiner score), type I collagen and plasma enzymes were assessed. At D63, early life activity significantly reduced histopathology scores (total, portal inflammation, steatosis, ballooning, but not lobular inflammation or fibrosis) and the number of rats affected. At D120, HF control scores were higher than in chow-fed controls, but the effect of activity was selective: early exercise reduced portal inflammation, steatosis, ballooning and fibrosis, but late activity affected only portal inflammation and ballooning. The chow-fed portal inflammation score was significantly less than all HF groups, but lobular inflammation was lower in the HF control group only. The fibrosis score in the HF early exercise and control chow group were lower than in the late exercise and sedentary HF groups, indicating that early life exercise was more effective than when activity was introduced later in life. Plasma biomarkers showed minor between-group differences. The retained effect on liver histopathology rat at D120 after only early life exposure activity suggests that timing of introduction of exercise is critical in reducing FLD scores and prevalence in children, young adults and possibly into adulthood.

Non-alcoholic fatty liver disease (NAFLD) is the most common cause of chronic liver disease in children. Also referred to as paediatric fatty liver disease (pFLD)^(1)^, research to date also suggests that the prevalence is increasing^(2)^ which is of major concern given the close association with development of both cirrhosis and cardiometabolic syndrome in adulthood. Prevalence is 3–10 % in the general paediatric population, and 60–70 % in those with metabolic diseases (Nobili, 2013^(3)^; Mencin, 2011^(4)^; Schwimmer, 2006^(5)^). NAFLD is characterised by accumulation of fat in the liver, termed hepatosteatosis, and can progress to non-alcoholic steatohepatitis (NASH), characterised by liver inflammation and hepatocyte ballooning, and in some cases to liver fibrosis and cirrhosis^(6)^. NAFLD represents a significant public health problem as not only is the prevalence of NASH higher in male children in subpopulations with high BMI, Asian or Hispanic ethnicity, diabetes or prediabetes, or panhypopituitarism^(7–9)^, but also because it is the most rapidly increasing liver disease causing hepatocellular carcinoma in patients younger than 40 years old awaiting liver transplantation^(10)^.

Children who are overweight or obese have a higher risk of liver cirrhosis as adults, leading to the suggestion that those with obesity tendency, or have any component of the metabolic syndrome, should be screened by ultrasonography and blood biomarker assessment^(11)^. However, neither these nor MRI-based assessments reliably discriminate NASH from FLD^(12)^, and prediction of progression from stable FLD to fibrosis is difficult^(1)^. Of the 17 % of all children in Western countries who are overweight or obese, 70–80 % have pFLD^(13,14)^; this is most likely because FLD is the hepatic expression of the metabolic syndrome, the prevalence of which also is very high in the young^(15,16)^. Although alterations in diet and physical activity (PA) can attenuate NAFLD, the success of such lifestyle alterations has been limited^(17)^.

Despite a lack of consensus whether pFLD represents the early stages of an age-progressive disease^(18)^, the increasing prevalence of pFLD may drive an increase in the disease burden of end-stage disease in adulthood. Various aspects limit accurate diagnosis of FLD in children, leading to calls for a better understanding of the pathogenesis of pFLD, which is less well defined than FLD in adults^(2)^. More explicit knowledge of how metabolic pathways are perturbed may direct interventions in reducing incidence in the young, as well as testing novel ways of attenuating or slowing progression of pFLD^(19)^. In managing the latter, gastroenterologists recommended PA 40 min/d (mean) on 5 d/week, along with reduction in sedentary (screen) time, although few randomised controlled trials have been conducted, and the knowledge gap of insufficient prescription of PA persists, possibly because in randomised controlled trials centred on childhood obesity, most children do not have NAFLD^(20)^.

One of the PA characteristics that may have an important effect on organ development and metabolic phenotype is the age at which additional activity is introduced. In our previous study, we used an animal model aiming to mimic a high-fat (HF) feeding environment in young children, where Sprague–Dawley male rats were fed a HF diet from weaning until adulthood (120 d old) and allowed exercise in addition to spontaneous cage activity for 37 d from weaning. We showed body composition, bone morphology and bone marrow molecular changes^(21)^, with persistent gene effects in bone marrow fat at least 60 d after cessation of the wheel exercise.

Using the same experimental approach, we tested the hypothesis that either early or late exercise, in the setting of a HF diet, would lead to differential effects on markers of FLD and collagen type 1 deposition as compared with sedentary animals fed either a control or HF diet.

## Materials and methods

### Study design

The experimental design has been described previously^(21)^. Ethical approval was granted by the University of Auckland Animal Ethics Committee (AEC001432). Animals were bred in-house by the University of Auckland Vernon Janson Animal Unit. Briefly, eighty weanling (23 day old) male Sprague–Dawley rats (*n* 20 per group) were numbered 1–80 and randomised (using an automatic number generator in Excel) and pair-housed in standard cages in a room maintained with 12: 12 h light/dark cycle, 21°C and ambient humidity, and *ad libitum* access to water. One group of rats were fed a standard chow diet (18 % energy content from fat, Diet 2018, Envigo) while the other three were fed a HF diet (45 % energy content from fat, D12451, Research Diets) from weaning. The composition of each diet is presented in Table 1, and the fatty acid profile of each diet is provided in online Supplementary Tables 1 and 2. Food and water were checked at regular intervals.

**Table 1.** Diet composition

Macronutrients	Chow diet	High-fat diet
	Global 18 % protein rodent diet	R12451 research diets
Protein, g %	18·6	24
Carbohydrate, g %	6·2	24
Fat, g %	44·2	41
Fat source	Soyabean oil	Lard, soyabean oil
Kcals from protein (%)	24	20
Kcals from fat (%)	18	45
Kcals from carbohydrate (%)	58	35
Kcals/g	3·1	4·73

**Fig. 1.**
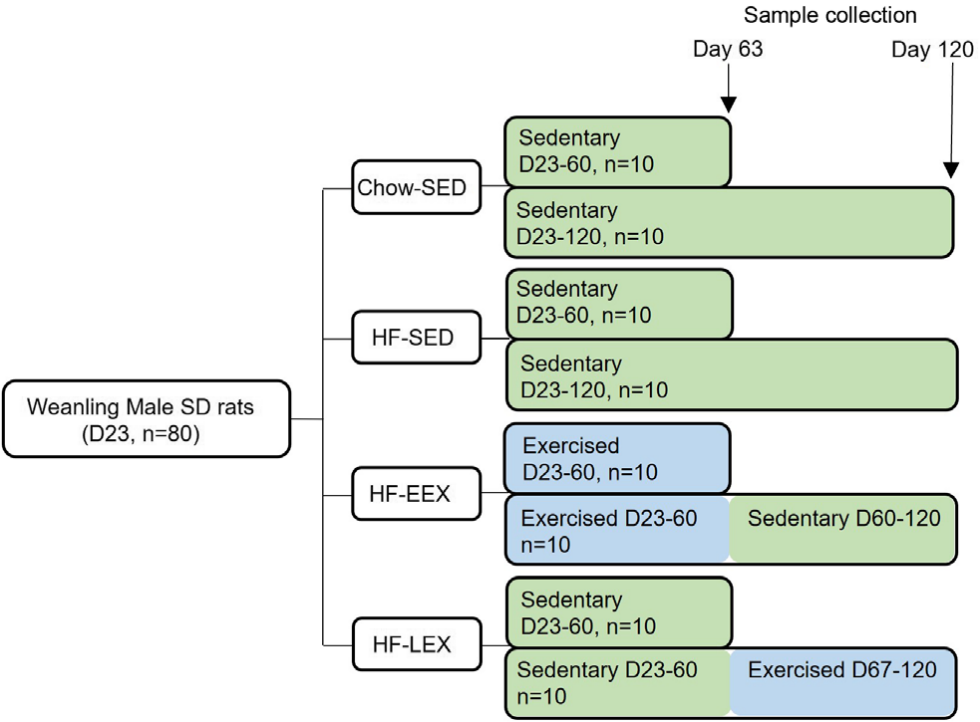
Summary of the study design. Eighty Sprague–Dawley (SD) weanling males were randomised to a chow diet or high-fat (HF) diet from Day 23 (D23) and housed in cages without a running wheel (sedentary – SED; Chow-SED and HF-SED). Two groups of HF-fed rats had access to a running wheel either between D23 and D60 (early exercise – EEX; HF-EEX) or from D67 to D120 (late exercise – LEX; HF-LEX). *N* 10 animals from each group were culled at D63 and D120.

The chow-fed group (Chow-SED) and one HF group (HF-SED) were limited to spontaneous cage activity. An early life exercise group was provided with an exercise wheel (Model 80859, Lafayette Instrument Company) from D23 to D60 (HF-EEX); a late exercise (HF-LEX) group had wheel access from D67 to 120. Half of each group (5 pairs, *n* 10) were culled at D63 and half at D120 for sampling. Wheel exercise data were recorded at 15 min intervals using dedicated monitoring software (Model 86065). Preliminary analyses of the exercise data confirmed that the rats in the HF-EEX group used the wheel preferentially during the dark period (18: 00–6: 00 h) with minimal activity during the day. For this reason, only dark period wheel exercise data were analysed. For the HF-EEX group, the wheels were removed from the housing cages after D63. The study design is summarised in Fig. 1. Animals were fasted overnight, anaesthetised using sodium pentobarbitone (60 mg/kg, IP) and culled by decapitation. Five pairs of rats from each group were culled at either D63 or D120. After the positive effect of EEX on liver histopathological scores was found in a preliminary study of D120 rats, the D63 liver samples were processed using identical protocols as detailed below.

### Plasma biomarkers

Plasma concentrations of insulin and leptin were analysed by commercially available rat-specific ELISA (90 060 and 90 040, respectively, Crystal Chem). The liver enzymes alanine aminotransferase (ALT), aspartate aminotransferase and alkaline phosphatase concentrations were measured as a measure of animal welfare and liver health using a Hitachi 902 autoanalyser (Hitachi High-Technologies Corporation).

### Liver histopathology scoring

Part of the right medial lobe of the liver was excised and placed in 4 % paraformaldehyde for 1 h at room temperature. Routine paraffin-embedded liver sections (5 µm) were collected 100 µm apart and stained with haematoxylin and eosin. Sections were blinded and ten fields of view from five non-consecutive sections per liver were assessed on two independent occasions to ensure consistency of scoring using the Kleiner NASH histological scoring system^(22)^. A grade for each of the five characteristics according to severity or extent of change between minimal/zero and severe changes detected using the following scoring system: steatosis (G0–G3), lobular inflammation (G0–G3), hepatocyte ballooning (G0–G2), portal inflammation (G0–G1) and fibrosis (G0–G4)^(22)^. The extent of fibrosis was performed based on stage 0, no fibrosis; stage 1, zone 3 fibrosis; stage 2, zone 3 and portal fibrosis; stage 3, zone 3 and portal fibrosis with bridging fibrosis; and stage 4, cirrhosis. The sum of the scores produced a total NAFLD score.

### Fibrotic area

In the Masson’s trichrome-stained slides (from the uneven numbered rats of each group, *n* 5 per group), histological fields per section were captured (Nikon, Eclipse E400). The digital images at 10× magnification were analysed using a colour threshold detection system developed in ImageJ (version 1.52a, NIH), to isolate the green-stained fibrotic tissue and quantify the fibrotic area occupied as a fraction of the total area of the five random fields (identified fibrotic area/total liver area) × 100.

### Immunohistochemical staining of collagen type I

Rabbit polyclonal to collagen type I antibody (Rockland 600-401-103) was used to quantitatively determine density of staining of collagen type I. Briefly, 5-µm liver sections were deparaffinised and rehydrated, and heat-induced epitope retrieval was performed in 10 mM sodium citrate buffer (pH6). Immunostaining was performed using the Novolink Polymer Detection System Kit (Novocastra, Leica Biosystems), following the manufacturer’s instructions. Sections were treated with peroxidase block for 5 min, incubated with protein blocking agent for 5 min, washed with Tris-buffered saline (pH 7·5), incubated overnight at 4°C with rabbit anti-collagen type I antibody (1: 500 dilution), washed again with Tris-buffered saline and incubated with anti-rabbit poly-HRP-IgG for 30 min at room temperature. Collagen type I was visualised with DAB chromogen staining. Sections were counter-stained with haematoxylin and examined under an optical microscope (Nikon, Eclipse E400). Ten random fields of view from each section were captured for image analysis from a total of twelve animals (*n* 3 per experimental group). The optical density of collagen type I quantified using ImageJ.

### Statistical analysis

Data are represented as mean values with their standard error of the mean grade for Kleiner characteristics, total NAFLD score, fibrotic area and collagen type I staining. After testing for normality, one-way ANOVA with Holm–Sidak correction was used to determine the significance of between-group differences. Statistical analysis was undertaken using SigmaPlot (Version 14.0; Systat Software). Statistical significance was defined as *P* < 0·05.

For histopathological analysis, the number of animals with obvious histopathological compromise, that is, equal to or greater than G3, G2, G1 for the first four liver histology scores listed above, and G2 and G3 for fibrosis, was expressed as a proportion of each treatment group and analysed using a generalised linear model for binomial data.

## Results

### Body composition, plasma biomarkers and physical activity

Fat and lean mass body phenotype data have been previously reported^(21)^ and are presented in online Supplementary Fig. 1 to support the pathological findings of the current study. Briefly, at D63 the HF-SED group body weight was greater than other groups, due to the higher fat mass and the fat/lean mass ratio; at D120, body weights were similar between HF-SED and HF-EEX groups and significantly higher than the HF-LEX and Chow-SED groups.

**Fig. 2.**
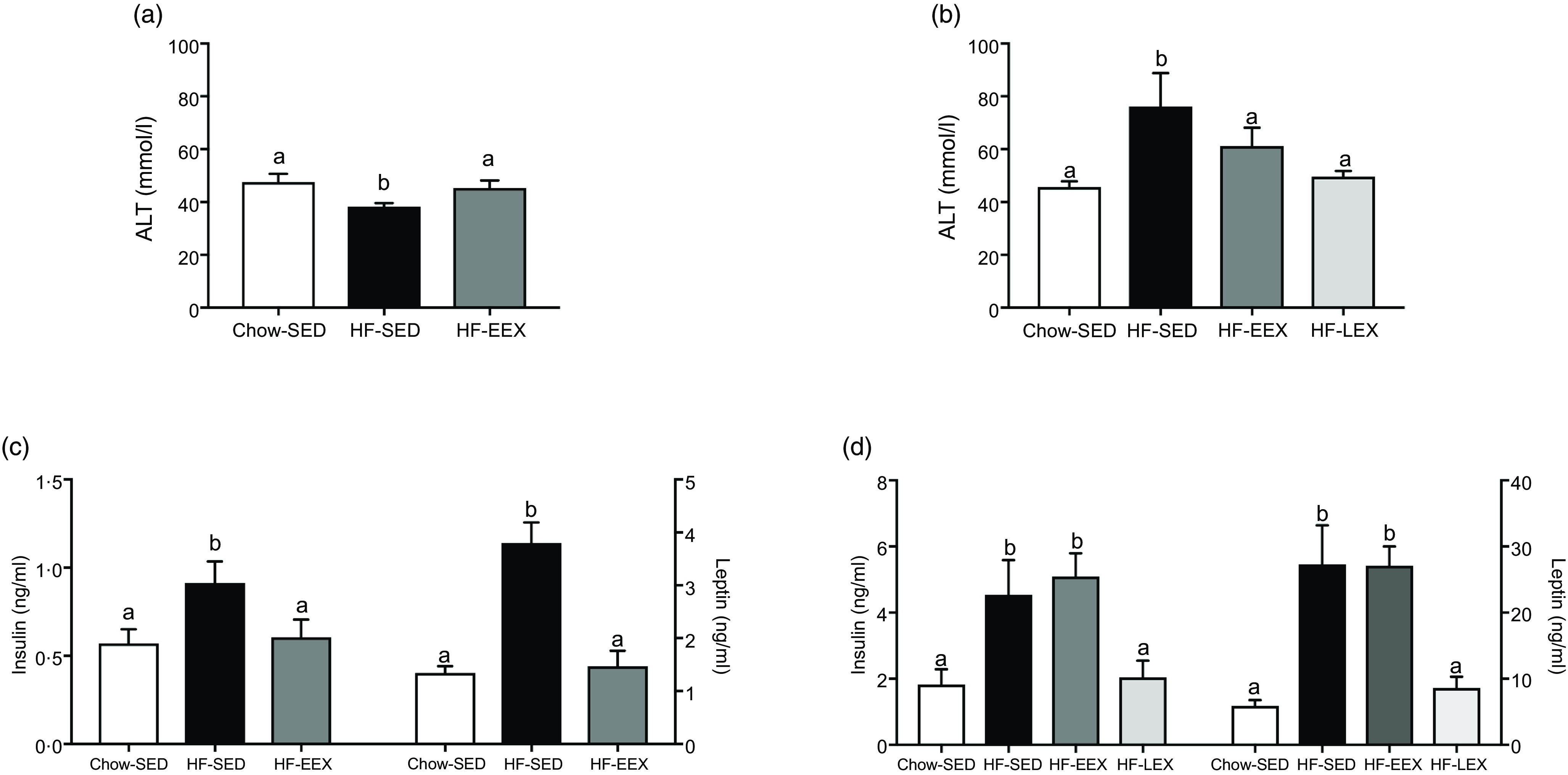
Plasma concentrations (mean values with their standard error of the mean) at D63 (left) and D120 (right) of (a), (b) alanine aminotransferase (ALT), and (c), (d) insulin and leptin. Significant intergroup differences (*P* < 0·05) determined by one-way ANOVA with Holm–Sidak correction are indicated by different letters. Data represent ten animals for Chow-SED and HF-EEX and ten animals for HF-SED.

Of the liver enzymes, only plasma ALT was significantly different between groups (Fig. 2(a)). At D63, ALT concentrations were significantly lower in the HF-SED group compared with Chow-SED and HF-EEX (*P* = 0·01 and 0·043, respectively; F value (–df) 39, 39) = 5·97). However, by D120 ALT was significantly greater in the HF-SED group than Chow-SED (*P* = 0·029; F value (df 37, 37) = 5·138) as shown in Fig. 2(b). There were no differences across groups for plasma aspartate aminotransferase or alkaline phosphatase concentrations (data not shown).

At D63, plasma insulin and leptin concentrations at D63 were significantly higher in HF-SED group as compared with the Chow-SED or HF-EEX group (*P* = 0·07 and < 0·001, and F value (df 39, 39) = 2·729 and (df 39, 39) = 16·79, respectively) (Fig. 2(c)). At D120, insulin and leptin concentrations were similar in HF-SED and HF-EEX groups and significantly higher compared with both HF-LEX and Chow-SED groups (F value (df 38, 38) = 7·123 and 15·548 for insulin and leptin, respectively) as presented in Fig. 2(d).

The PA has been published elsewhere^(21)^. Briefly, the HF-EEX group was allowed running wheel activity from D23, and there was an increase in PA from day 23 at 988·59 (sem 347·05) m/cage per night to 9971·97 (sem 1933·34) m/cage per night at D60. Similar to the HF-EEX animals, the HF-LEX animals exhibited a gradual increase in distance covered (from 1377 (sem 254) m/cage per night to an average of 9907 (sem 2550) m/cage per night). Most activity (∼98 %) occurred during the dark phase of the 24 h light/ dark cycle.

### Liver histopathology scores

**Fig. 3.**
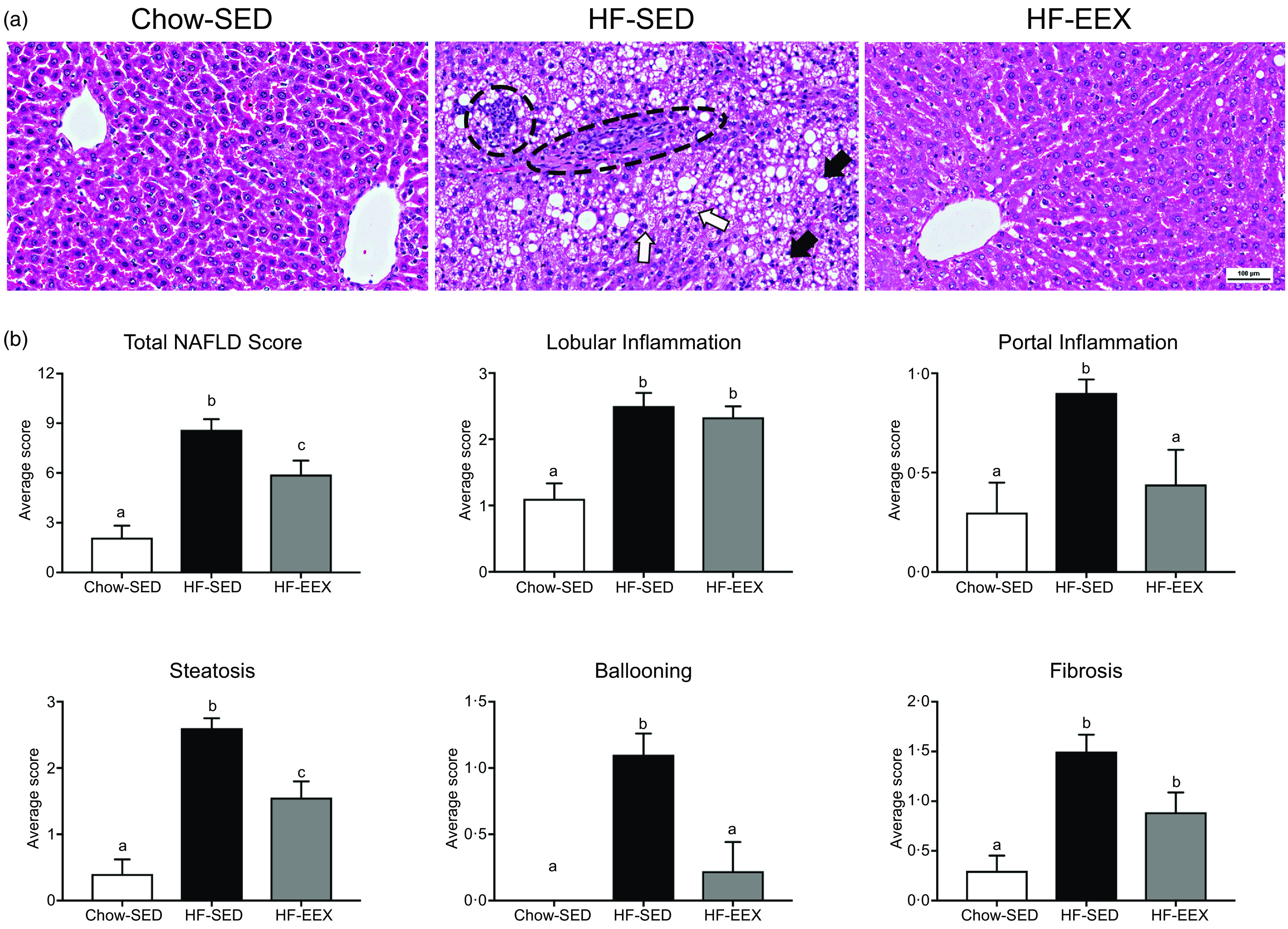
Liver histopathology grading for 63-d-old rats. (a) Representative images of liver haematoxylin and eosin-stained sections from rats exposed to high-fat (HF) diet and limited to cage activity only (HF-SED), HF diet with access to early life wheel exercise (HF-EEX) or to chow diet (Chow-SED). Dashed ovals represent lobular inflammation, white arrows represent ballooned hepatocytes, black arrows represent micro- and macrovesicular steatosis. Scale bar represents 100 μm. (b) Mean values with their standard error of the mean total NAFLD score and the five components. Statistical significance was determined using one-way ANOVA with Holm–Sidak correction. Significant intergroup differences (*P* < 0·05) are indicated by different letters. Data represent ten animals for Chow-SED and HF-EEX and ten animals for HF-SED.

At D63, five characteristic features of NAFLD and the NAFLD total score were significantly higher in the HF-SED group as compared with the Chow-SED group (Fig. 3). The HF-EEX group had a lower score than those of the HF-SED group for all measures except for lobular inflammation and fibrosis.

At D63, Chow-SED rats showed no evidence of steatosis, lobular inflammation, ballooning or fibrosis, but several had portal inflammation (Fig. 3). Overall, the dietary effect was significant for all scored components of the Kleiner scoring (ranging between *P* < 0·001–0·046) except for stage 3 fibrosis as shown in Table 2. The proportion of rats with histological evidence of steatosis and portal inflammation changes was significantly lower in the HF-EEX than in the HF-SED group (*P* = 0·008 and 0·017, respectively), although the fraction of EEX group with these five indices was approximately 2–3-fold lower than in the HF-SED group (Fig. 3 and Table 2).

**Table 2.** The proportion (%) of each of the groups (*n* 10) with obvious-severe scores of steatosis (grade ≥G3), lobular inflammation (G3), ballooning (G2), portal inflammation (G1) and fibrosis (G2, and G3). Subgroup analyses are presented

Group	Cull Day 63			*P*		Cull Day 120				*P*		
	Chow-SED	HF-SED	HF-EEX	HF-SED *v*. HF-EEX	Overall	Chow-SED	HF-SED	HF-EEX	HF-LEX	HF-SED *v*. HF-EEX	HF-SED *v*. HF-LEX	Overall
Steatosis	0	70	20	0·008	<0·001	10	70	40	50	0·174	0·359	0·037
Lobular inflammation	0	70	40	0·115	<0·001	30	90	60	70	0·112	0·255	0·036
Ballooning	0	30	10	0·198	0·046	0	90	40	50	0·015	0·044	<0·001
Portal inflammation	30	90	50	0·017	0·002	20	100	60	90	0·010	0·230	<0·001
Fibrosis grade 2	0	55	20	0·061	0·002	10	90	50	80	0·044	0·528	<0·001
Fibrosis grade 3	0	5	10	0·615	0·49	0	50	0	40	0·003	0·653	0·002

**Fig. 4.**
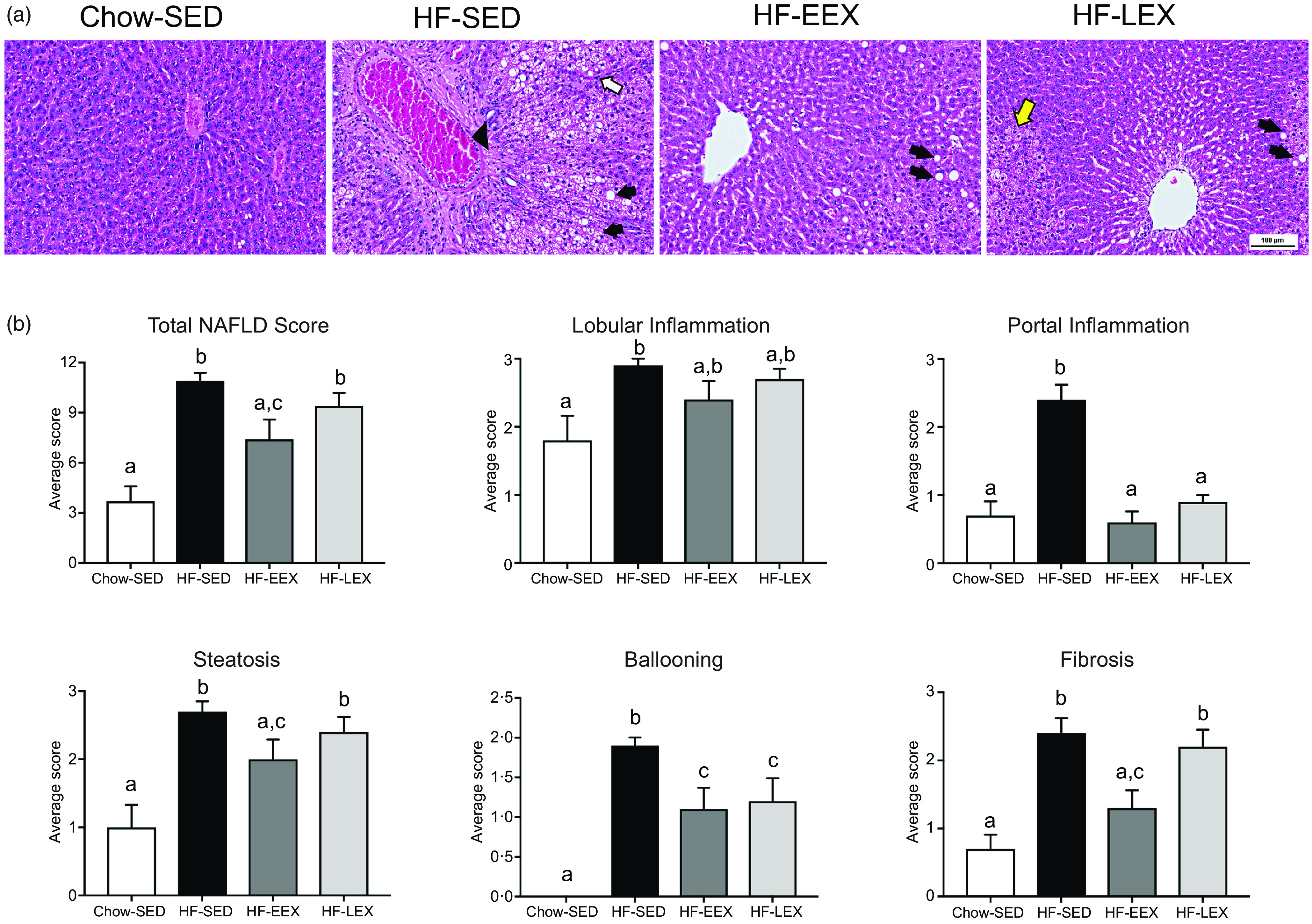
Liver histopathology grading for 120-d-old rats. (a) Representative images of liver sections from rats exposed to high-fat (HF) diet and limited to cage activity only (HF-SED), HF diet with access to early life wheel exercise (HF-EEX), HF diet with access to later life wheel exercise (HF-LEX) or to chow diet (Chow-SED) under bright field microscopy stained with haematoxylin and eosin. Scale bar represents 100 μm. In HF-SED, the white arrow indicates necro-inflammatory foci, the black arrowhead indicates zone 3 fibrosis with fibrotic septa formation and black arrows indicates micro- and macro-vesicular steatosis. In HF-EEX, black arrowheads indicate mild micro-vesicular steatosis. In HF-LEX, black arrowheads indicates micro- and macro-vesicular steatosis. The yellow arrow indicates lobular inflammation. (b) Histopathology grade for total and five components of fatty liver disease (FLD) in four groups of 120-d-old rats. Data represent the mean values with their standard error of the mean from ten rats per group. Statistical significance was determined using a one-way ANOVA with Holm–Sidak correction. Significant intergroup differences (*P* < 0·05) are indicated by different letters. Data represent ten animals per experimental group.

At D120, the total and contributing scores of the HF-SED group were significantly higher than in the Chow-SED groups as shown in Fig. 4. The Chow-SED group had a lobular inflammation score lower than that of the HF-SED but not HF-EEX or HF-LEX groups. Portal inflammation grade was similar in HF-EEX, HF-LEX and Chow-SED groups, all being significantly less than in HF-SED. The score of fibrosis was significantly lower in Chow-SED and HF-EEX than in HF-SED and HF-LEX groups.

At D120, the proportion of animals with histopathological changes was significantly less in the Chow-SED than in any of the three HF groups (*P* < 0·001–0·037). Several rats in the Chow-SED group had obvious inflammation and one had stage 2 fibrosis. Compared with the HF-SED group, the number of rats was significantly (*P* = 0·044–0·003) less in the HF-EEX group for all histological characteristics except steatosis and lobular inflammation; in the HF-LEX group, the count was significantly less (*P* = 0·044) in the case of ballooning only (Table 2).

### Fibrotic area and collagen type I

**Fig. 5.**
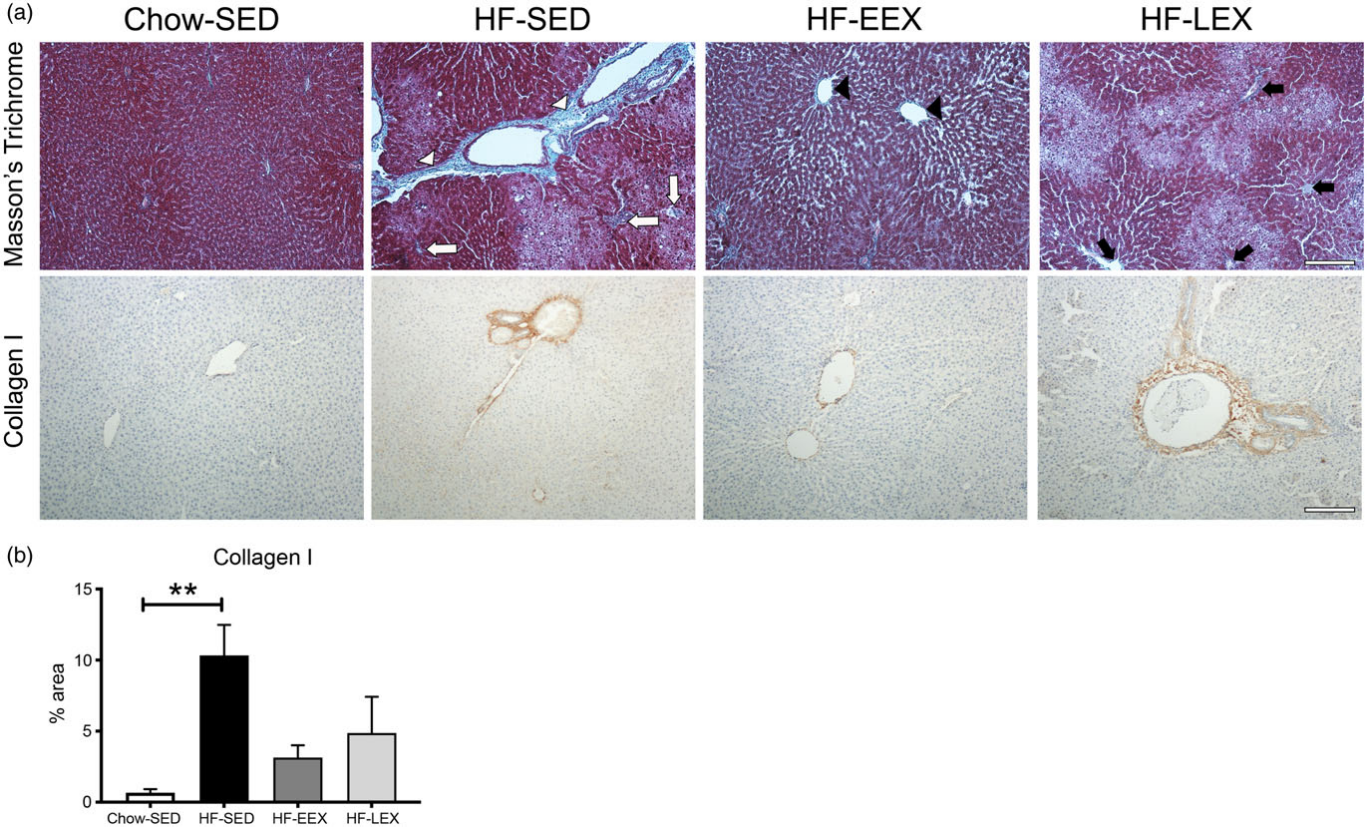
Liver fibrosis. (a) Representative images of liver sections from D120 rats from each group stained with either Masson’s trichrome (top row) or immunolabelled for collagen type I (bottom row). (Top row) in the Masson’s trichrome stained sections: HF-SED white arrowheads indicate thick fibrous septa (bridging septa), white arrows indicate pericellular and perisinusoidal fibrosis; HF-EEX black arrowheads indicate zone 3 fibrosis, and in HF-LEX black arrows indicate zone 3 perisinusoidal/portal and periportal fibrosis. (b) Percentage area (%) stained for collagen type I. Statistical significance was determined using a one-way ANOVA with Holm–Sidak correction where **P < 0·01 compared with Chow-SED. Data represent analysis from ten random fields of view from three animals per experimental group.

Liver fibrosis of each group assessed by Masson’s trichrome staining and collagen type I immunostaining are shown (Fig. 5(a)), with area positively staining for collagen type I quantified in Fig. 5(b). The HF diet induced significant portal fibrosis, as indicated by the differences between HF-SED sections relative to Chow-SED (*P* = 0·007). This effect was ameliorated in both HF-EEX and HF-LEX treatment groups, which were not statistically different from Chow-SED.

## Discussion

Our main finding is that compared with other groups, the EEX group had a significantly lower FLD histopathology score and lower prevalence of moderate–severe histological FLD changes in adulthood (D120). This was 60 d after the cessation of voluntary wheel exercise in the EEX group, indicating that the induced liver phenotype was retained long after the PA environment, despite the dietary environment remaining unchanged. The protective effects of EEX on liver pathology were not paralleled by changes in other whole body phenotypic features (D120 body composition, leptin and insulin concentrations) as shown above and as reported in earlier studies^(6,21)^. Thus, retention of the EEX effects on liver histopathology, independent of other biomarkers, may be of a similar nature to those on bone marrow gene expression profile^(21)^ which had significant between-group differences and retained a ‘memory’ of up- or down-regulation of regulator genes affecting some metabolic pathways disturbed by intake of HF diets. This is consistent with alterations in the early life environment resulting in development programing effects on chronic disease risk across the life course. The prenatal and postnatal factors known to influence pFLD have been reviewed^(2)^.

We did not confine scrutiny to the combined NAFLD histopathology score in case each of its five components was influenced independently by EEX. We also included prevalence (number of rats affected) with moderate or worse grades of each of the five liver histopathological features, so that data of differences between individuals and groups would inform on obvious FLD without possible confounding through misinterpretation of subtle changes (lower grades) of each component. This was because NASH is progressive, and differences in milder forms of NAFLD and NASH are unclear^(6)^. Also, moderate to severe changes in phenotype is a good foundation for studying associations with other biomarkers, comparison across experiments and species, and the impact of different environmental factors on assessment of FLD ranging from steatosis through lobular/portal inflammation, and progression to its more severe and prognostically significant forms (NASH). The latter is characterised by progression of ballooning and fibrosis, but the activity score used to separate mild through severe NAFLD^(22)^ is founded on adult and paediatric human (not rodent) data.

The severity and prevalence of histopathology change at D120 suggested that the chronic insult of the HF diet had resulted in histopathological features typical of NASH, and the **s**tage 2 fibrosis in HF diet rats already present at D63 was confirmed with staining for the presence of type I collagen in serial sections in three animals per treatment group.

The D120 group mean scores and prevalence of histopathological abnormalities demonstrated that EEX was more effective in limiting liver lesions than was LEX, except in the case of hepatocyte ballooning. The LEX group had access to the running wheel for 53 d, compared with 38 d for the EEX group. Over these periods, the EEX and LEX groups gradually increased daily activity (to 9971 and 9907 m/cage per night, respectively). However, distance decreased to 3254 m/cage per night in the latter part of the LEX period^(21)^, which might have contributed to the histopathology scores being significantly more favourable in the EEX than the LEX group at D120. The LEX group was also exposed to the HF diet for a much longer period, and HF diets are known to have an inhibitory effect on voluntary PA^(23)^. Further, the period of PA acclimatisation from weaning to D60 in HF-EEX and HF-SED groups may have accustomed the EEX rats to undertake more spontaneous cage PA (playing, climbing, etc.) after D63, but we cannot not confirm this as were unable to assess spontaneous cage activity.

A notable feature of PA is that it selectively influenced some histological characteristics and not others. For instance, at D63 in the HF-EEX group the PA had a significant effect of suppressing steatosis, ballooning, portal inflammation, but not lobular inflammation. In the HF-LEX group, the PA had a significant effect on only portal inflammation and ballooning.

The NAFLD scoring demonstrated that individual rats in the CHOW-SED were not immune from FLD, or its progression. At D63, the CHOW-SED group prevalence of all FLD features was zero, except for the 30 % prevalence of portal inflammation. By D120, several CHOW-SED rats had histopathological changes (except ballooning and stage 3 fibrosis), with mean scores that were numerically higher than at D63, except for hepatocyte ballooning (zero at both stages). The results might be explained by differing individual propensity for these rats to develop FLD, likely from (combinations of) genetically inherited variation in control mechanisms of energy and fat metabolism (including food intake) and expected variability in PA across a control population of animals.

That almost all FLD features had responded to voluntary PA by D63 was likely due to the anti-inflammatory effect of PA on the HF diet-induced inflammation in rodents, as indicated by others^(24,25)^. The HF-EEX group had retained its significant amelioration effect on NAFLD total and component histopathology scores, except lobular inflammation, by D120. Most striking is that the positive effects of lower (although not statistically significant) score and lower prevalence of fibrosis in the EEX group at D63 were preserved at D120, 60 d after early life wheel activity ceased and in the face of continued exposure to the HF diet. This is an important finding, since it portends that early life PA of particular types (e.g. number of cycles, balance of aerobic/anaerobic activity) may have a lasting effect on attenuating severity and prevalence of liver fibrosis. That said, we do not know how long this effect would have lasted into adulthood beyond D120, and further study is required because the degree and rate of change in fibrosis are the single most important histopathological feature determining risk of progression to NAFLD in young people.

Despite the NAFLD histopathological findings, we did not determine the fraction of total liver volume that was normal/abnormal. This could differ between individuals and groups because of variation in birth size, liver weight gain, and inflammatory and regenerative responses to the HF diet, and effects of PA. If such differences did exist between the D63 and D120 whole liver responses, it might account for the anomalous lower mean plasma ALT concentration in the HF-SED group at D63, compared with the expected higher ALT at D120 after longer exposure to both HF diet and lack of PA. The PA undertaken is also highly variable. Rats’ inclination to move is suppressed by HF diet^(26)^ and which was also demonstrated by another study where voluntary wheel (both daily and 4 week cumulative) distances in adult male mice on a control diet exceeded by 2·5-fold those on HF diet^(25)^. Various rodent studies^(25,27–30)^ show that exercise generally decreased ALT plasma concentrations, and that lack of PA increased them, similar to that occurring in human adults with NAFLD^(31,32)^. We can explain the lower ALT concentration in the HF-SED group at D63 only based on possible effect of a higher fraction of metabolically competent liver tissue in HF-SED at D63 than D120, and of higher spontaneous cage PA in the HF-SED group than the HF-EEX group PA from combined cage and wheel PA. During the course of pFLD, ALT shows variability^(33)^ and is a poor marker of disease activity^(2)^.

Current research into FLD in young people is by necessity dominated by the search for possible treatments for established disease in young adults. The understanding of the pathogenesis of pFLD is incomplete, and it remains unclear pFLD is indeed the initial form of the NAFLD disease which progresses with age into and through adulthood^(18)^. The only ‘treatment’ available for NAFLD is weight loss^(10)^, but the loss required to alter NAFLD status and outcomes is not agreed upon^(34)^. In children with NAFLD, even 10 % weight loss after diet modification and medium intensity exercise for 1 year did not lead to a marked reduction in markers of disease^(6)^ ^(35)^. Due to limited randomised controlled trial data from lifestyle interventions in pFLD, prescription of PA by clinicians was discouraged^(36)^.

In our study, the EEX from D22 to D60 resulted in body weight difference (of only 4·4 %) from the HF-SED group, but despite reversion to HF-diet phenotype by D120, the effects on bone marrow and liver tissues were retained. We have outlined that persistent transcriptional control of some candidate pathways possibly accounted for the retained effect on bone metabolism^(21)^ and suggest that a similar ‘memory’ mechanism might account for the retention of the effect of EEX on liver histopathology grades long after the EEX stimulus had been withdrawn. Due to the bleak outlook in most populations for the future health of people afflicted with advanced NAFLD^(10)^, we suggest that the strategic application of EEX might contribute to prevention or attenuation of FLD in children, if further work shows that EEX programming-induced metabolic changes and specific histopathological changes are maintained into mid-late adulthood.

We studied only male rats due to constrained resources, and our results cannot be directly extrapolated to females given the potential sex-specific differences that exist in the prevalence, risk factors, fibrosis and clinical outcomes of NAFLD (Lonardo, 2019^(37)^). The requirement to house male rats in pairs for animal welfare reasons (to avoid stress responses) precluded obtaining individual data that could have allowed study of individual proclivity to liver abnormality. The total PA volume consists of wheel activity and spontaneous cage activity. Because each of these might affect the other, characterisation of total PA ‘volume’ associated with between-group biomarker differences was not quantified. Given the findings, this is an important matter requiring further study in the exercise prescription area, since the PA that produces the desirable effects may be far less than commonly assumed, equivalent more to a low-moderate than a moderate-high intensity. The low compliance with the latter and with endurance training in both older and younger human sub-populations is well documented.

Early life exercise in the setting of NAFLD has been subject to only scant investigation. We conclude from our study of small groups of rats that voluntary wheel PA in early life had a significant capacity to attenuate histopathological and plasma NAFLD biomarkers within weeks of its introduction and to attenuate features of NAFLD in adulthood, long after the additional voluntary PA ceased. Voluntary wheel PA beginning at a later age was less effective than EEX in suppressing NAFLD scores and prevalence.

## Supplementary material

For supplementary material accompanying this paper visit https://doi.org/10.1017/S0007114522002562. click here to view supplementary material
