# Supplementary material for: Voluntary physical activity in early life attenuates markers of fatty liver disease in adult male rats fed a high-fat diet
Source: Br J Nutr. 2022 Aug 11;129(10):1667–76. doi: 10.1017/S0007114522002562 (PMC10116184; doi:10.1017/S0007114522002562)
Supplement: Supplementary file 1 [file S0007114522002562sup001.docx]

**Supplementary Table 1**. Fatty Acid composition of the standard chow (Teklad Global 18% Protein Rodent Diet).

| **Fatty Acids** | **Composition (%)** |
| --- | --- |
| C16:0 Palmitic | 0.7 |
| C18:0 Stearic | 0.2 |
| C18:1ω9 Oleic | 1.2 |
| C18:2ω6 Linolenic | 3.1 |
| C18:3ω3 Linolenic | 0.3 |
| Total Saturated | 0.9 |
| Total Monosaturated | 1.3 |
| Total Polyunsaturated | 3.4 |

**Supplementary Table 2.** Fatty acid composition of the high fat chow (R12451 Research Diets). The fatty acid composition is based in 177.5 g lard and 25g soybean oil. Information provided by Research Diets. The percentage (%) of saturated, monosaturated and polyunsaturated fats is also provided.

| **Fatty Acids** | **Based on 175 g lard & 25 g soybean oil** |
| --- | --- |
| C10, Capric | 0.1 |
| C12, Lauric | 0.2 |
| C14, Myristic | 2.1 |
| C15 | 0.1 |
| C16, Palmitic | 36.8 |
| C16:1, Palmitoleic, n-9 | 2.5 |
| C17 | 0.7 |
| C18, Stearic | 19.8 |
| C18:1, Oleic, n-9 | 64.1 |
| C18:2, Linoleic | 56.2 |
| C18:3, Linolenic | 4.2 |
| C20, Arachidic | 0.4 |
| C20:1 | 1.2 |
| C20:2 | 1.4 |
| C20:3, n-6 | 0.2 |
| C20:4, Arachidonic, n-6 | 0.5 |
| C22, Behenic | 0.1 |
| C22:5, Docosapentaenoic, n-3 | 0.2 |
| Total | 190.7 |
| Total Saturated | 60.2 (31%) |
| Total Monosaturated | 67.7 (35.5%) |
| Total Polyunsaturated | 62.8 (13.1%) |
